# Assessment of subclinical left ventricular systolic and diastolic dysfunction in patients with type 2 diabetes mellitus under follow-up at Tikur Anbessa specialized hospital, Ethiopia: a case-control study

**DOI:** 10.1186/s12872-024-03850-x

**Published:** 2024-04-06

**Authors:** Tigist Seleshi, Theodros Alemneh, Dufera Mekonnen, Demu Tesfaye, Sura Markos, Yitagesu Getachew, Konno Taddese, Senbeta Guteta

**Affiliations:** 1 Department of Internal Medicine, Division of Cardiology, College of Health Sciences, Addis Ababa University, Addis Ababa, Ethiopia; 2 Department of Internal Medicine, Division of Endocrinology, College of Health Sciences, Addis Ababa University, Addis Ababa, Ethiopia; 3 Department of Internal Medicine, Division of Cardiology, Adama Hospital Medical College, Adama, Ethiopia; 4 Department of Internal Medicine, Division of Cardiology, School of Medicine, Hawassa University, Hawassa, Ethiopia; 5 Department of Internal Medicine, Division of Cardiology, Yekatit Hospital Medical College, Addis Ababa, Ethiopia

**Keywords:** Diastolic dysfunction, Left ventricular systolic dysfunction, Diabetic adults, Ethiopia

## Abstract

**Background:**

Individuals with diabetes mellitus are at increased risk of cardiovascular diseases, which in turn are the most common cause of morbidity and mortality in the diabetic population. A peculiar feature of cardiovascular diseases in this population is that they can have significant cardiac disease while remaining asymptomatic. There is a paucity of data regarding subclinical cardiac imaging features among diabetic adults in Africa, particularly in Ethiopia. This study was conducted to compare the magnitude and spectrum of left ventricular systolic and diastolic dysfunction among asymptomatic type 2 diabetic adults versus a normotensive, non-diabetic control group and to evaluate the determinants of left ventricular diastolic and systolic dysfunction.

**Methods:**

This was a case-control study conducted at Tikur Anbessa specialized hospital, Addis Ababa, Ethiopia. A standard transthoracic echocardiography was done for all study participants with type 2 diabetes mellitus and their normotensive and non-diabetic controls. Structured questionnaires were used to collect demographic and clinical characteristics and laboratory test results. Statistical analysis was done using the SPSS 25.0 software. The data was summarized using descriptive statistics. Bivariate and multivariate analysis was performed to determine the association between variables and echocardiographic parameters. The strength of statistical association was measured by adjusted odds ratios and 95% confidence intervals, with significant differences taken at *p* < 0.05.

**Results:**

We analyzed age- and sex-matched 100 participants in the study (diabetic) group and 200 individuals in the control group. Left ventricular systolic and diastolic dysfunction were significantly more prevalent among diabetic adults than their sex and age matched controls. Among diabetic individuals, ages of 60 years and above, dyslipidemia, use of Metformin and Glibenclamide, high serum triglyceride level, presence of neuropathy and use of statins correlated significantly with the presence of left ventricular diastolic dysfunction. Chronic kidney disease and neuropathy were determinants of left ventricular systolic dysfunction.

**Conclusion:**

Left ventricular systolic and diastolic dysfunction were significantly more prevalent among diabetic patients than their sex- and age-matched controls in our study. We recommend early screening for subclinical left ventricular dysfunction, especially in the elderly and in those with chronic kidney disease, dyslipidemia, and microvascular complications such as neuropathy.

## Background

The prevalence of diabetes is increasing globally at an alarming rate. In 2013, it was projected that 300 million people would be diagnosed with diabetes by the year 2030, but the current prevalence in 2022 has already surpassed this number by 100 million [1]. In a large-scale survey conducted in Ethiopia in 2015, the prevalence of diabetes and pre-diabetes was determined to be 5.6% and 5.4%, respectively [2]. Micro- and macrovascular complications of diabetes mellitus are major determinants of morbidity and mortality of patients. Of these, cardiovascular complications account for the majority of the disease burden in relation to diabetes [1, 2].

The risk of developing cardiovascular complications and heart failure is by far higher in diabetic patients as compared with non-diabetic ones. Diabetes was found to be associated with an increased risk of heart failure in patients with non-obstructed coronary arteries. The earliest description of diabetic cardiomyopathy was from an autopsy study that evaluated the vascular and myocardial findings of diabetic patients that had glomerulosclerosis. Postmortem study of four patients with no additional risk factors showed cardiomegaly and signs of heart failure with no major coronary obstruction. Based on the findings of intramural arterial thickening and narrowing, micro-angiopathy related to diabetes was considered to be the culprit. This has led to a better understanding of the pathogenesis behind diabetic-induced microvascular and myocardial dysfunction leading to heart failure [3, 4].

Both systolic and diastolic dysfunction are observed frequently in diabetic patients. As seen in the Framingham study, the rate of development of heart failure was five times and twice as high in diabetic women and men, respectively, as compared to non-diabetic patients. Among diabetic patients with heart failure, 30% had diastolic dysfunction, considered the earliest sign of heart failure in diabetic individuals [5]. Diastolic dysfunction is seen to be associated with poor glycemic control. Microvascular dysfunction, renin-angiotensin-aldosterone system imbalance, collagen formation and degradation imbalance, impaired calcium transport, and interstitial accumulation of glycosylation products are all possible mechanisms of diastolic dysfunction in diabetic patients. Diabetes, along with aging, hypertension, and atrial fibrillation, contributes significantly to the pathogenesis and prognosis of diastolic dysfunction [6].

Various studies have tried to identify the factors associated with diastolic dysfunction in type 2 diabetic patients. An observational study that included about 49,000 patients identified that poor glycemic control was associated with a more severe diastolic dysfunction. Each 1% increase in hemoglobin A1C (HbA1C) was linked to an 8% increase in the risk of heart failure. These findings were also confirmed in a single, small-scale study of type 1 diabetic patients with diabetic neuropathy [7, 8]. Small-scale case-control studies from the U.K., Turkey and Australia identified that while systolic function was similar, diastolic function was markedly impaired in diabetic patients. Poor diabetic control, advancing age, treatment with Metformin and a longer duration of diabetes were significantly associated with echocardiographic abnormalities of diastolic dysfunction while the presence of left ventricular dysfunction, and treatment with insulin and angiotensin-converting enzyme inhibitors (ACEIs) were found to be protective [9–11].

There is a paucity of data regarding the subclinical cardiac imaging features among diabetic adults in Africa, particularly in Ethiopia – the second most populous nation in the continent and one with a high number of its citizens being pre-diabetic or diabetic [2]. This study was conducted to compare the magnitude and spectrum of left ventricular systolic and diastolic dysfunction among asymptomatic type 2 diabetic adults versus a normotensive, non-diabetic control group and to evaluate the determinants of left ventricular diastolic and systolic dysfunction.

## Methods

### Study setting

This was a case control study conducted from June – October 2022 at Tikur Anbessa Specialized Hospital (TASH), Addis Ababa, Ethiopia.

### Study design

This was a case-control study with the source population being all patients with type 2 diabetes mellitus aged 40 years and above and under follow up at the study hospital for at least 6 months and having no prior history of cardiac illness or symptoms. (No prior history of cardiac illness is defined as a patient with no previous diagnosis, treatment or follow up for a cardiac compliant. That was extracted from the diagnosis list on the electronic medical recording and during history).

Diagnosis of Type 2 Diabetes Mellitus: HBA1c>6.5%, RBS>200 mg/dl, FBS> 126mg/dl, on two separate set of tests.

These symptoms include paroxysmal nocturnal dyspnea, orthopnea, dyspnea, chest pain (anginal type), body swelling not attributable to other causes, etc with a combination of symptoms given precedence over solitary symptoms. We enrolled 100 individuals and a further 200 age- and sex-matched controls using a convenience sampling method. The control group were normoglycemic, normotensive surgical patients, aged 40 years and above presenting to the study hospital for non-cardiac complaints.

Patients who were found to have a combination of symptoms that suggest cardiovascular disease were excluded.

### Data collection

After obtaining informed consent, structured questionnaires were used to collect demographic and clinical characteristics and laboratory test results from patient interviews and charts. A standard transthoracic echocardiography was also performed on all study participants, using the same equipment (Vivid 9, GE equipped with tissue Doppler imaging and a transducer of 1.5 – 2.5 MHz). Each recorded measurement was the mean of three measurements (Table 1). If both left ventricular systolic and diastolic dysfunction were identified in the same participant, then the presence of diastolic dysfunction was not included in the analysis as it could be due to the mere presence of systolic dysfunction even in the absence of diabetes [12].

**Table 1:** Operational definitions

Parameter	Operational definition (Ref. [13])
LVSD - All measurements are averages of three measurements - If any one of the three measurements was positive then it was considered enough to define LV systolic dysfunction except in patients with LVH where the presence of LVEF ≤ 50% is mandatory to define LVSD^a^	LVEF < 50% (biplane Simpson’s method)
	S’ wave velocity < 8 (TDI) (Ref. [14])
	Septal MAPSE < 7 mm Lateral MAPSE < 8 mm (Ref. [15])
LVDD is defined as any measurement which fell outside of the depicted values for E/A ratio and deceleration time (normal ranges). - A combination of 3 positive findings were considered to define diastolic dysfunction.	Normal values for E/A ratio and deceleration time
	E/A ratio = 0.8 - 1.5
	Deceleration time = 140 – 240 ms
	Septal E/e’ ratio >15
	e’ Lateral < 10 (age < 55), < 9 (age 55 - 65) < 8 (age > 65)
Grade I LVDD	E/A < 0.8, Reduced e’ for age (Ref. [16])
Grade II LVDD	E/A > 0.8, Reduced e’ for age, Dilated left atrium (Ref. [16])
Grade III LVDD	E/A > 1.5, Reduced e’ for age E-wave DT < 140 ms

Key: *EF* ejection fraction, *LVSD* left ventricular systolic dysfunction, *LVH* left ventricular hypertrophy, *LVEF* left ventricular ejection fraction, *LVDD* left ventricular diastolic dysfunction, *MAPSE* Mitral annular peak systolic excursion, *E/A* ratio- early diastolic flow velocity/late diastolic transmitral flow velocity, *E/e’* ratio of early diastolic mitral inflow velocity to early diastolic mitral annulus velocity , *e’* mitral annular velocity

^a^LVH by itself was found to be associated with reduced MAPSE despite normal EF and Global longitudinal strain

Subclinical Diastolic and systolic dysfunction means an Echocardiographic finding without symptoms.

Echocardiographic measurements were taken by blinded operator and the interpretation was done by other members of the team following the standard definitions.

Strain Analysis is demonstrated to have superior sensitivity and specificity for LV systolic dysfunction. The setup to do strain analysis is not available in our country.

### Data analysis

Statistical analysis was done using the SPSS 25.0 software. The data were summarized using descriptive statistics. Bivariate and multivariate analysis was performed to determine the association between variables and echocardiographic parameters. The strength of statistical association was measured by adjusted odds ratios and 95% confidence intervals, with significant differences taken at *p* < 0.05.

## Results

### Socio-demographic characteristics

The study enrolled 300 individuals: 100 cases and 200 controls. The two groups were sex- and age- matched. The majority of the participants were 50 - 70 years of age (Table 2).

**Table 2:** Socio-demographic characteristics of the study participants

Variable		Total (*n*)	Cases (%)	Controls (%)
Age (years)	40 – 50	85	29 (29%)	56 (28%)
	50 – 60	92	31 (31%)	61 (30.5%)
	60 – 70	89	31 (31%)	58 (29%)
	70 – 80	30	8 (8%)	22 (11%)
	> 80	4	1 (1%)	3 (1.5%)
Duration of diabetes since diagnosis	< 5 years	23	23 (23 %)	-
	5 - 10 years	30	30 (30 %)	-
	>10 years	47	47 (47 %)	-
Sex	Female	168	56 (56%)	112 (56%)
	Male	132	44 (44%)	88 (44%)
Complications arising from diabetes	Retinopathy	8	8 (8%)	-
	Nephropathy	33	33 (33%)	-
	Neuropathy	27	27 (27%)	-
	Peripheral arterial disease	3	3 (3%)	-
	Stroke/TIA	2	2 (2%)	-
Laboratory results	LDL (mg/dl)	105.9 ± 39.7	105.9 ± 39.7	-
	HbA1C (%)	8.7 ± 1.6	8.7± 1.6	-
	Triglyceride (mg/dl)	155.5 ± 87.1	155.5 ± 87.1	-
Comorbidity	Dyslipidemia	46	26 (26%)	20 (10%)
	Hypertension	47	47 (47%)	-
	CKD	9	9 (9%)	-
	HIV	7	7 (7%)	8 (4%)
	Cholelithiasis	40	-	40 (20%)
	BPH	50	-	50 (25%)
	Nephrolithiasis	17	-	17 (8%)
	Malignancy	30	-	30 (15%)
	COPD	20	-	20 (10%)
	Hip Fracture	20	-	20 (10%)
	Thyroid Nodule	10	-	10 (5%)

Key: *TIA* Transient ischemic attack, *LDL* low-density lipoprotein, *HbA1C* Hemoglobin A1C, *CKD* Chronic kidney disease, *HIV* Human immunodeficiency virus, *BPH* Benign prostatic hyperplasia, *COPD* Chronic obstructive pulmonary disease

### Cases

The mean duration of diabetes among the study group was 10.9 years. The majority of patients (47%) had a long period since diagnosis of diabetes, defined as more than 10 years with the remaining 23% and 30% of participants having spent 5 years and 5 - 10 years respectively since diagnosis. Various comorbidities were identified in the study group, with hypertension being the most common (47%). The participants in this group were screened for micro- and macrovascular complications of diabetes mellitus, with nephropathy the most commonly identified complication (33%) (Tables 2, 3 and 4).

**Table 3:** Laboratory results of the study group versus left ventricular diastolic function status

Variables in the study group	LVDD	Normal diastolic function	Total
Age (years)	57 ± 10.1	51 ± 8.72	56 ± 10.12
LDL (mg/dl)	107.5 ± 41.7	98.7 ± 28.3	105.9 ± 39.7
HbA1C (%)	8.8 ±1.69	8.3 ± 1.5	8.7 ± 1.6
Triglyceride (mg/dl)	161.2 ± 90.3	129.7 ± 66.9	155.5 ± 87.1
Ejection Fraction (%)	58.59 ± 7.6	61.6 ± 5.90	59 ± 7.4
Duration of diabetes (years)	11.1 ± 7.45	9.8 ± 9.0	10.9 ± 7.74

Key: *LVDD* left ventricular diastolic dysfunction, *LDL* low density lipoprotein, *HbA1C* Hemoglobin A1C

**Table 4:** Laboratory results of the study group versus left ventricular systolic function status

Variables among cases	LVSD	Normal systolic function	Total
Age (years)	57.12 ± 11.21	55.94 ± 10.0	56.12 ± 10.12
LDL (mg/dl)	87.09 ± 30	108.26 ± 40	105.9 ± 39.7
HbA1C (%)	9.28 ± 1.46	8.65 ± 1.67	8.7 ± 1.6
Triglyceride (mg/dl)	153.27 ± 57.7	155.85 ± 90.34	155.5 ± 87.1
Ejection Fraction (%)	42.54 ± 3.85	61.2 ± 4.61	59 ± 7.4
Duration of diabetes (years)	15.09 ± 7.2	10.38 ± 7.68	10.9 ± 7.74

Key: *LVSD* left ventricular systolic dysfunction, *LDL* low density lipoprotein, *HbA1C* Hemoglobin A1C

The study group were on multiple medications, with Metformin being the most commonly used drug (48%) followed by a combination of metformin and sulfonylureas (32%). Only 4 patients were on sodium glucose transport protein 2 inhibitors (SGLT2Is) (one patient on canagliflozin and 3 on dapagliflozin). A total of 48% of patients were taking insulin either in combination with other oral hypoglycemic agents or alone. About 53% of patients were taking ACEis or angiotensin receptor blockers (ARBs). Of these, 45% were on Enalapril, 5% on Losartan and 3% on Candesartan. Nearly 85% of patients were on statins with 88% of those receiving statins were taking Atorvastatin and the remaining12% either on rosuvastatin or simvastatin. The remaining 15% were not taking any type of statin drugs.

### Controls

Of the various causes of admissions to the hospital among the control group, benign prostatic hyperplasia (BPH) (25%), cholelithiasis (20%), and malignancies (15%) accounted for the majority of admissions. Dyslipidemia was identified as comorbidity in 10% of the control group. All the patients included in the control group had normal fasting blood glucose levels and were normotensive. Their mean ejection fraction was 61.78% (± 4.95%). The remaining laboratory results had normal findings. A comparison of echocardiographic abnormalities of the two groups is shown in Table 5.

**Table 5:** Baseline echocardiographic parameters of the study group vs control group

Variables		Total (*n*)	Cases (%)	Controls (%)
LVDD	Grade I	101	51 (51%)	50 (25%)
	Grade II	37	27 (27 %)	10 (5%)
	Grade III	5	4 (4%)	1 (0.5%)
	Normal diastolic function	158	18 (18%)	140 (70%)
LVSD	LV EF < 50%	14	11 (11%)	3 (1.5%)
	LVEF ≥ 50%	286	89 (89%)	197 (98.5%)
Lateral e’	Reduced	51	51 (51%)	-
	Normal	49	49 (49%)	200
Septal e’	Reduced	60	60 (60%)	-
	Normal	40	40 (40%)	200

Key: *LVDD* left ventricular diastolic dysfunction, *LVSD* left ventricular systolic dysfunction, *LVEF* left ventricular ejection fraction. *Lateral e’* lateral S wave velocity, *Septal e’* septal S wave velocity

### Echocardiographic comparisons

Of the 100 diabetic patients, 11 had a left ventricular ejection fraction < 50% with 89 participants having normal left ventricular systolic function (LVSF) as measured by all parameters. Three had moderate left ventricular systolic dysfunction (LVSD) eight of the patients have a mildly reduced ejection fraction. Three of the patients with mild LVSD had regional wall motion abnormalities (anterior, anteroseptal, and apical wall hypokinesis). Reduced lateral and septal S wave velocities were seen in 51% and 60% of the participants, respectively (Table 5).

When compared to the controls, there was a significantly higher prevalence of diastolic dysfunction and systolic dysfunction. The rate of systolic dysfunction in the cases was higher when compared to the control group despite the smaller sample size (OR: 0.13, 95% CI 0.03 – 0.6, *p* = 0.01). Similarly higher diastolic dysfunction was seen in sub-group analysis of the study group than in the controls (grades I – II). Although there was no difference in grade III diastolic dysfunction between the two groups, it should be noted that its prevalence in both groups is very low (4% in cases and 0.5% in the control group), limiting our ability to compare it among the two groups (Table 6).

**Table 6:** Multivariate analysis of echocardiographic findings

Variables	Case (*n* = 100)	Control (*n* = 200)	OR* (*95% CI*)*	*P* value
Grade I diastolic dysfunction	51 (51%)	50 (25%)	5.9 (3.1 - 7.1)	0.0001
Grade II diastolic dysfunction	27 (27%)	10 (5%)	2.9 (1.1 - 7.3)	0.0001
Normal diastolic function	18 (18%)	140 (70%)		-

### Determinants of LVSD and LVDD

Among diabetic individuals, ages 60 years and above, dyslipidemia, use of Metformin and Glibenclamide, a high serum triglyceride level, presence of neuropathy and use of statins correlated significantly with the presence of left ventricular diastolic dysfunction. Chronic kidney disease and neuropathy were determinants of left ventricular systolic dysfunction, as seen by reduced left ventricular ejection fraction (Table 7).

**Table 7:** Multivariate analysis of echocardiographic findings among the study (diabetic) group

Diastolic dysfunction (*n* = 82)	OR* (*95% CI*)*	*P* value
Combined use of Metformin and Sulfonylureas	0.147 (0.028 – 0.763)	0.02
High triglyceride level	0.385 (0.154 – 0.961)	0.04
Dyslipidemia	0.356 (0.132 – 0.960)	0.04
Dyslipidemia	0.264 (0.098 – 0.713)	0.009
Atorvastatin use	0.375 (0.114 – 1.26)	0.033
Neuropathy	0.048 (0.004 - 0.552)	0.015
Reduced left ventricular ejection fraction (< 50%)		
Chronic kidney disease	0.193 (0.04 - 0.920)	0.039
Neuropathy	0.257 (0.071 – 0.929)	0.038



Echo Image [26]: Representative of diastolic function assessment

## Discussion

Our study showed that left ventricular systolic and diastolic dysfunction were significantly more prevalent among diabetic adults than their sex and age matched controls. Hypertension was the most common comorbidity in our study population (47%). The Framingham study showed that hypertension had an additive adverse effect on the left ventricular volume, relaxation, diastolic function, and systolic function of the left ventricle [5]. But hypertension was not found to be significantly correlated with left ventricular systolic and diastolic function in our study.

In our study, the prevalence of diastolic dysfunction was 82% among the diabetic patients as compared to 30% in the control group. As compared to other studies in low-income settings, our findings showed a higher prevalence. A study conducted in Nigeria with a similar sample size to our study, found that 65% of the diabetic patients had diastolic dysfunction, compared to 3.3% in the control group. This disparity can be explained by the relatively older age of patients included in our study (56 years vs 50.8 years) and having a longer duration of diabetes (10.9 years vs 3.4 years in in Dodiyi-Manuel et al’ study from Nigeria) [1]. Case-control studies with larger cohorts from the U.S. confirm that diabetic individuals had a significantly higher number of subclinical left ventricular dysfunction [17–19]. A large study of 751 diabetic adults conducted in Italy discovered a similar higher prevalence of diastolic dysfunction as our study, with approximately 60% of the participants having left ventricular diastolic dysfunction [20].

We identified a reduced left ventricular ejection fraction in about 11% of the diabetic sub-group of our study. These findings mimicked those of other studies. In a case-control study conducted in Nigeria, 15.6% of participants had an LV ejection fraction of 55%, compared to 4% in the control group. The authors used a cut-off ejection fraction of less than 55%, which may have overestimated the prevalence [21]. Another study done to evaluate biventricular systolic dysfunction in patients with type 2 diabetes compared 26 cases with 126 control participants. Diabetes showed an independent association with severely decreased biventricular function and an LV ejection fraction of < 30%. In 15.4% of the patients, there was also associated right ventricular dysfunction [17].

More advanced imaging modalities, such as speckle tracking and strain analysis, show a higher prevalence of subclinical LVSD than 2D echocardiography. The use of dobutamine/exercise stress echocardiography has demonstrated that diabetic patients have a higher prevalence of LV dysfunction [22–24]. In a study that evaluated longitudinal and radial strain of the left ventricle in 32 diabetic patients and 32 control subjects, there was significantly lower longitudinal strain with preserved LV ejection fraction in type 2 diabetic individuals, which was explained by normal radial strain compensation [24]. We were not able to do strain analysis because of a lack of access. But we were able to evaluate the lateral and medial S’ wave velocities. Tissue Doppler imaging revealed that 51 - 60% of our study population had significantly lower lateral and septal s-wave velocities. Systolic wave velocity is considered to be a surrogate marker of early left ventricular dysfunction and a measure of longitudinal systolic function. and was found to be associated with LV ejection fraction. A study that evaluated subclinical LVSD and glycemic control in asymptomatic type 2 diabetic patients with preserved LV ejection fraction identified that mean S’ wave velocity was inversely and independently associated with high HgbA1c after adjustment for age, diabetes duration, and body mass index [25].

Our study also showed that among diabetic individuals, ages 60 years and above, dyslipidemia, use of Metformin and Glibenclamide, high serum triglyceride level, presence of neuropathy and use of statins correlated significantly with the presence of left ventricular diastolic dysfunction. We identified that grade I diastolic dysfunction was significantly associated with ages above 60 years, combined use of metformin and sulfonylurea and a high serum triglyceride level. Dyslipidemia was also associated with both grade I and grade II LVDD. Use of Atorvastatin was significantly associated with Grade II LVDD.

Our findings are similar to most other studies which have concluded that, in particular advanced age and use of Metformin being significant predictors of diastolic dysfunction. A small-scale study of 70 patients showed that serum LDL levels, a high HbA1c level, and the patient's age were significantly associated with LV dysfunction [9]. While many studies showed factors like duration of diabetes, poor glycemic control as adverse predictors and use of ACEIs as having a protective effect against LVDD and LVSD, such findings could not be replicated in our study [7, 9, 10]. This could be due to the smaller sample size that we enrolled.

Chronic kidney disease and neuropathy were determinants of left ventricular systolic dysfunction in our study. Other studies have confirmed these findings, with additional adverse factors identified being poor glycemic control and advanced age [10, 11].

The comparison between the case and control groups in our study showed that diabetes was independently associated with a higher prevalence of left ventricular grade I and II diastolic dysfunction, and a reduced ejection fraction.

The limitations of our study were our relatively smaller sample size and the unavailability of natriuretic peptide tests (brain natriuretic peptide BNP/N-terminal pro b-type natriuretic peptide NT-ProBNP) and advanced imaging techniques like speckle tracking.

## Conclusion

Our study showed that left ventricular systolic and diastolic dysfunctions were significantly more prevalent among diabetic patients when compared to normoglycemic, normotensive controls. Among the diabetic sub-group, ages of 60 years and above, dyslipidemia, use of Metformin and Glibenclamide, neuropathy and use of statins predicted the occurrence of left ventricular diastolic dysfunction. Chronic kidney disease and neuropathy were also found to be associated with left ventricular systolic dysfunction. The presence of diabetes and its comorbidities is associated with subclinical left ventricular systolic and diastolic dysfunction. Therefore, similar studies should be done in low-resource settings to devise screening programs aiming to detect subclinical left ventricular dysfunction early, especially in the elderly and in those with predisposing comorbidities.

## Data Availability

All study data can be made available from the corresponding author upon a reasonable request.

## Abbreviations

ACEI: Angiotensin converting enzyme inhibitor
ARBs: Angiotensin receptor blockers
BMI: Body Mass Index
BP: Blood pressure
BPH: Benign prostatic hyperplasia
CKD: Chronic kidney disease
CVD: cardiovascular diseases
COPD: Chronic obstructive pulmonary disease
E/A: ratio- early diastolic flow velocity/late diastolic transmitral flow velocity
E/e’: ratio of early diastolic mitral inflow velocity to early diastolic mitral annulus velocity
EF: Ejection fraction
HbA1C: Glycosylated hemoglobin level
HIV: Human immunodeficiency virus
Lateral e’: lateral S wave velocity
LDL: low density lipoprotein
LV: Left Ventricle
LVDD: Left ventricular diastolic dysfunction
LVEF: Left ventricular ejection fraction
LVH: left ventricular hypertrophy
LVSD: Left ventricular systolic dysfunction
MAPSE: Mitral annular peak systolic excursion
Septal e’: septal S wave velocity
SGLT2Is: Sodium glucose transport protein 2 inhibitors
TAPSE: Tricuspid annular peak systolic excursion
TASH: Tikur Anbessa Specialized Hospital
TIA: Transient ischemic attack
Type 2 DM: Type 2 Diabetes Mellitus
